# A Comparative Study of Grain Refining of Al-(7–17%) Si Cast Alloys Using Al-10% Ti and Al-4% B Master Alloys

**DOI:** 10.3390/ma16072867

**Published:** 2023-04-04

**Authors:** Agnes M. Samuel, Ehab Samuel, Victor Songmene, Fawzy H. Samuel

**Affiliations:** 1 Département des Sciences Appliquées, Université du Québec à Chicoutimi, Chicoutimi, QC G7H 2B1, Canada; 2 Department of Mechanical Engineering, École de Technologie Supérieure (ÉTS), Montréal, QC H3A 1K3, Canada

**Keywords:** grain refining, Al_3_Ti, AlB_2_, undercooling, Ti-Si interaction, microstructure

## Abstract

The present article addresses solidification parameters, and includes analyses of the macrostructure and microstructure in the light of the results obtained from the thermal analysis, from which it is possible to conclude that undercooling (T_S_) and recalescence (T_R_) temperatures increase with the initial increase in titanium (Ti) concentration. If the concentration reaches approximately 0.25%, a rapid decrease in these temperatures is observed. Thereafter, the temperatures increase again with the further increase in Ti concentration, and eventually become constant. These temperatures also vary depending on the superheating and casting temperature. The ∆T parameter (i.e., T_S_ − T_R_) decreases with the Ti concentration and, from a concentration of around 0.20% Ti, this parameter becomes zero. The grain size decreases with the Ti concentration. If the concentration exceeds about 0.20%, the grain size becomes the minimum. Another parameter to be considered is the interaction between the grain refiner and the traces of other metals in the base Al alloy. For example, Al-4%B can react with traces of Ti that may exist in the base alloy, leading to the reaction between boron (B) and Ti to form TiB_2_. Grain refinement is achieved primarily with TiB_2_ rather than AlB_2_, or both, depending on the Ti content in the given alloy.

## 1. Introduction

Grain refinement in aluminum−silicon (Al-Si) casting alloys is usually assessed by the presence of titanium (Ti) and boron (B). Since the 1980s, thermal analysis has established itself as an important alternative means for determining the degree of grain refinement, and also to predict the degree of modification of eutectic silicon. Grain refining is considered one of the most important liquid metal processing processes for aluminum (Al) alloys. Three different types of grain morphology are possible: columnar, twin columnar, and equiaxed. It is well known that an equiaxed grain structure provides uniform mechanical properties and reduces hot tearing, second phase distribution and microporosity on a fine scale [1,2,3].

Grain refining in aluminum alloys aims to increase the number of crystallization sites of the pro-eutectic phase (α-Al phase) and to avoid columnar growth. In order to have a fine scale grain size, the most widely practiced method is to present effective nuclei in the liquid metal using Al-Ti-B grain refiners, which usually contain active seeds such as Al_3_Ti, TiB_2_, AlB_2_, or (Al,Ti)B_2_. Thermodynamic studies suggest that these latter particles convert to TiB_2_, so that the titanium diffuses into the (Al,Ti)B_2_ particles while the aluminum diffuses out, resulting in the formation of TiB_2_ [4,5,6].

### 1.1. Thermal Analysis

**Figure 1:**
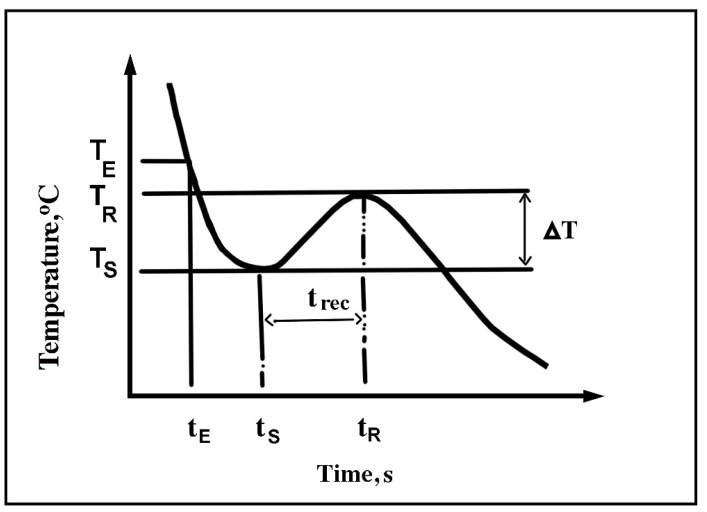
Effect of different parameters on the start of the solidification of dendrites of α-aluminum [9].

The solidification of any alloy or metal can be accompanied by some phenomena [6,7]. First, as the solidification rate is not infinitesimal (i.e., perfectly balanced), supercooling (∆TS) or “undercooling” [8,9,10] is possible (Figure 1). Solidification does not begin at the equilibrium temperature (T_E_) but at the supercooling temperature (T_S_). Thereby,

(1) $$\mathrm{Undercooling}\;(\Delta \mathrm{Ts})=T_{E}-T_{S}=(K_{1}+K_{2})\;\overset{•}{T}$$

where:

- ∆T_S_: undercooling (°C);
- T_E_: temperature at equilibrium (°C);
- T_S_: temperature at undercooling (°C);
- K_1_: constant as a function of solidification rate (s);
- K_2_: constant as a function of alloying (s);
- $\overset{•}{T}$: solidification rate (°C/s).

Thereafter, depending on different parameters (i.e., concentration of certain alloying elements), a recalescence is possible. During this phenomenon, the temperature of the alloy increases slightly to a maximum before falling again. This maximum temperature is called the “recalescence temperature” (T_R_). The latent heat emitted during the onset of solidification, when the alloy reaches temperature T_S_, partly explains this phenomenon. In addition, each of the temperatures (T_E_, T_S_, and T_R_) are associated with the corresponding time (t_E_, t_S_, t_R_). In addition, in the literature, parameters ΔT and trec are found. ΔT is equal to the difference between the recalescence temperature and the supercooling temperature (ΔT = T_R_ − T_S_). As for trec, it is equal to the difference between t_R_ (the time related to the temperature of recalescence) and t_S_ (the time related to the supercooling temperature). Thus, t_rec_ = t_R_ − t_S_.

### 1.2. Grain Nucleation

**Figure 2:**
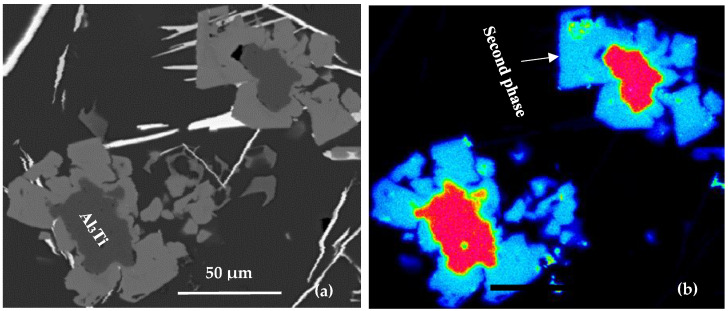
Precipitation of other phases or α-Al on the Al_3_Ti particles in Al-Si alloys: (**a**) Backscattered electron image, and (**b**) X-ray image of Ti in Al_3_Ti.

The theory of grain nucleation is based on the fact that nucleation is possible due to the addition of particles such as TiB_2_, AlB_2_, and (Ti,Al)B_2_ through a master alloy, as shown in Figure 2. Cibula [11] was the first to propose that the presence of boron or carbide particles can act as a seed site in liquid aluminum. The carbides are formed by the reaction of the residual carbon of the alloy with the added Ti, thus forming TiC. For the description of this theory, the discussion will be focused mainly on the effect of boron particles. 

The addition of B particles can, in fact, act as a germination agent. With Ti concentrations below 0.15%, boron particles or, more often, groups of B particles, on which dendrites enriched in Ti germinate, are found in the center of the grains. On the other hand, other information indicates that germination does not necessarily take place from these B particles, which are weak germination sites, at least lower than Al_3_Ti. First, there is no consistency between the crystal lattice of TiB_2_ and that of α-Al. Then, in the master alloys, B particles are found at the grain boundaries, while Al_3_Ti are located in the middle of the grain [12,13]. The studies of Samuel et al. [14] confirmed that boron particles are weak seed sites, because they are pushed back to the grain boundaries when there is no Ti in the solution in the aluminum. This phenomenon suggests that there is a good discontinuity between the lattice of the B particles and that of α-Al. In addition, the B particles require a certain degree of supercooling for the alloy to crystallize, whereas alloys containing Al_3_Ti do not [15]. 

### 1.3. Poisoning of Grain Refining

The elements dissolved in the liquid metal have great importance for the refining of grains. They limit the growth of the solid as they have to divide between the solid and the liquid. The final grain size of the cast product largely depends on the growth restriction imposed by the solute elements, which is well represented by the parameter Q = m·C_0_·(K − 1), with m being the liquidus gradient, C_0_ the composition of the liquid pool, and k the partition coefficient of the dissolved body between the solid and the liquid. In addition, these elements facilitate the columnar-to-equiaxed transition. However, these same elements can also act during the nucleation step by weakening or destroying the nucleation centers (poisoning of TiB_2_ by Si).

The studies of Canales et al. [16] and Farahany et al. [17] prove that Al-Si alloys that have more than 7 wt% Si respond poorly to grain refinement (or poisoning) by Al-Ti-B type master alloys. The latter are rendered ineffective or less effective in the presence of a large percentage of Si. Some other investigators have indicated that silicon poisons Al-Si alloys containing 3 wt% Si, where the “poisoning” effect of Si can be discussed in terms of its effect on the energy balance required for nucleation. The catalytic efficiency, f (Θ), of a substrate is given by the following formula:

f(Θ) = (2 + cos Θ) (1 − cos Θ)^2^/4 (2)

where Θ is the contact angle between the core and the substrate. Based on the classical model for heterogeneous nucleation, the smaller the value of f(Θ), the greater the catalytic efficiency of the substrate. Some investigators have mentioned that the value of f(Θ) can be adversely affected by the segregation of Si at the particle/liquid metal interfaces [18]. 

### 1.4. Objectives

The main purpose of this paper is to study grain refinement by Ti or B, as well as the different effects of the presence or increase in the concentration of these elements in A356.2 and A390.1 alloys. Thus, the objectives will be investigated through the following:

1. Analysis of the effect of increasing the concentration on the grain size. One of the particularities of Ti is to further reduce the size of the grains, compared with other elements. Several variables affect grain refining. The different variables that will be studied specifically are the refiner concentration, as well as the overheating and casting temperature. The solidification rate remains identical (≈1 °C/s) for all of the tests.
2. Study the different effects of increasing the grain refining concentration on solidification using thermal analysis. The latter makes it possible to study different phenomena and parameters related to (during) solidification.
3. Study the different effects of increasing the concentration of the added grain refiner on the microstructure. If there is an impact on the microstructure, there is necessarily an influence on the mechanical properties.
4. The introduction of AlB_2_ in the Al-4% B form in alloys containing traces of Ti leads to the reaction between B and Ti to form TiB_2_ which is a poor grain refiner.

## 2. Experimental Procedure

Table 1 lists the chemical composition of the A356.2 and A390.1 alloys used in the present work.

**Figure 3:**
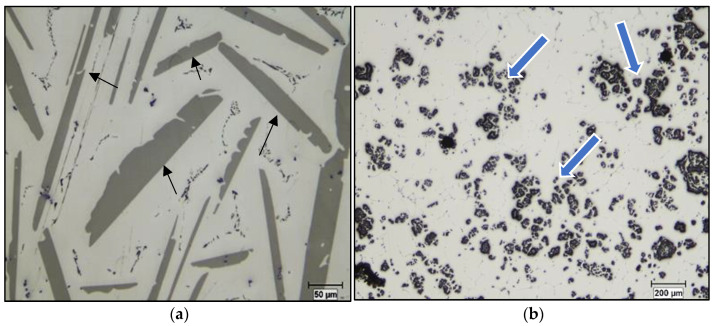
Optical micrographs showing the distribution of: (**a**) Al_3_Ti (black arrows) and (**b**) AlB_2_ particles (blue arrows) in Al-10%Ti and Al-4%B master alloys, respectively.

**Figure 4:**
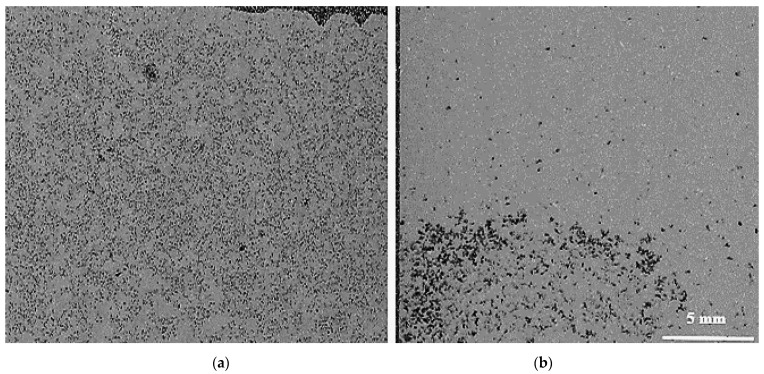
(**a**) Uniform distribution of Al_3_Ti particles with continuous mechanical stirring, (**b**) Sedimentation of Al_3_Ti particles to the bottom of the crucible with no mechanical stirring.

The received alloy ingots were cut into pieces of 650 g. One of these pieces was then placed in a SiC crucible and melted using an electrical furnace. During heating, the required Ti and B concentrations were added to the molten sample in the form of Al-10%Ti and Al-4%B master alloys, respectively (see Figure 3). When the melt reached the casting temperature, stirring was conducted for 15 min. The molten sample was poured immediately after this delay. If the sample was superheated to 950 °C and then poured at 750 °C, stirring was only done for 10 min at 950 °C and 750 °C. Mechanical stirring made it possible to adequately distribute the Ti and B charges throughout the molten metal. Mechanical stirring was particularly important for this system due to the difference between the density of the used alloy (2685 kg/m^3^) and that of the Al_3_Ti intermetallics (3368 kg/m^3^) [19]. To examine the effect of holding time at the required temperature, mechanical stirring was continued until the liquid metal was poured into the graphite mold [20] as shown in Figure 4.

**Figure 5:**
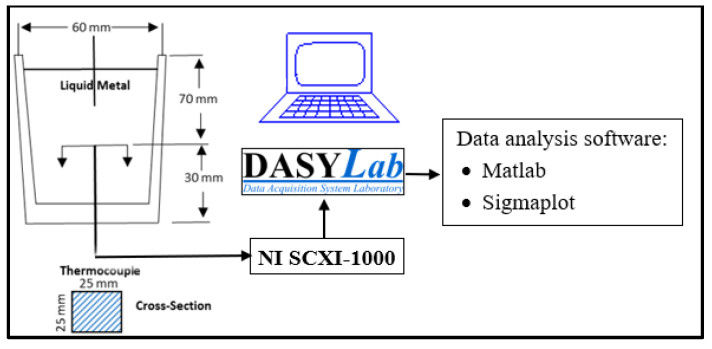
Schematic diagram of the thermal analysis set-up used in the present work.

For casting, a graphite cylindrical mold (dimensions are approximately 100 mm in height and 60 mm in diameter) was preheated to 600 °C. A few moments (approximately 10 s) before casting the sample, the mold was removed from the furnace. A type K thermocouple (chromel/alumel) was inserted through a hole in the bottom center of the mold for data on sample solidification (temperature versus time), which was collected using the data acquisition system shown in the schematic of Figure 5. The sampling rate was 50 readings/second. Data were collected until the temperature of the casting reached approximately 450 °C. The solidification rate was about 0.85 °C/s. To investigate the effect of holding time on the grain size, mechanical stirring was continued during this period.

In order to evaluate the significance of Ti addition on the tensile properties of the 356 alloy, ingots of the 356 alloy were melted in a 40 kg capacity SiC crucible using an electrical furnace. At 750 °C, measured amounts of Ti in the form of the (Al-10%Ti) master alloy were added progressively in increasing content to the molten metal that was degassed using pure argon. For each Ti concentration, 10 tensile bars were cast in a preheated metallic mold (ASTM B-108 type), preheated at 450 °C. The cast tensile bars were solutionized at 540 °C for 8 h, quenched in warm water at 60 °C, stored at room temperature for 24 h, and then aged at 155 °C for 5 h, followed by air cooling. All heat-treated tensile bars were pulled to fracture using an MTS servo-hydraulic machine at a strain rate of 4 × 10^−4^ s^−1^.

The microstructures of the selected samples were studied using various electron microprobe analysis techniques. The backscattered electron or “backscatter” mode made it possible to obtain an image of the microstructure, whereas dispersive X-ray or “EDS” and wavelength dispersive spectroscopy “WDS” were used to identify and determine the chemical composition of the different phases. For grain size measurements, the samples were etched using a solution composed of 66% HNO_3_, 33% HCl, 1% HF. The line intercept method was applied to measure the grain size. In each case, more than 150 grains were measured.

## 3. Results and Discussion

### 3.1. Solidification Aspects for the A356.2 Alloy

**Figure 6:**
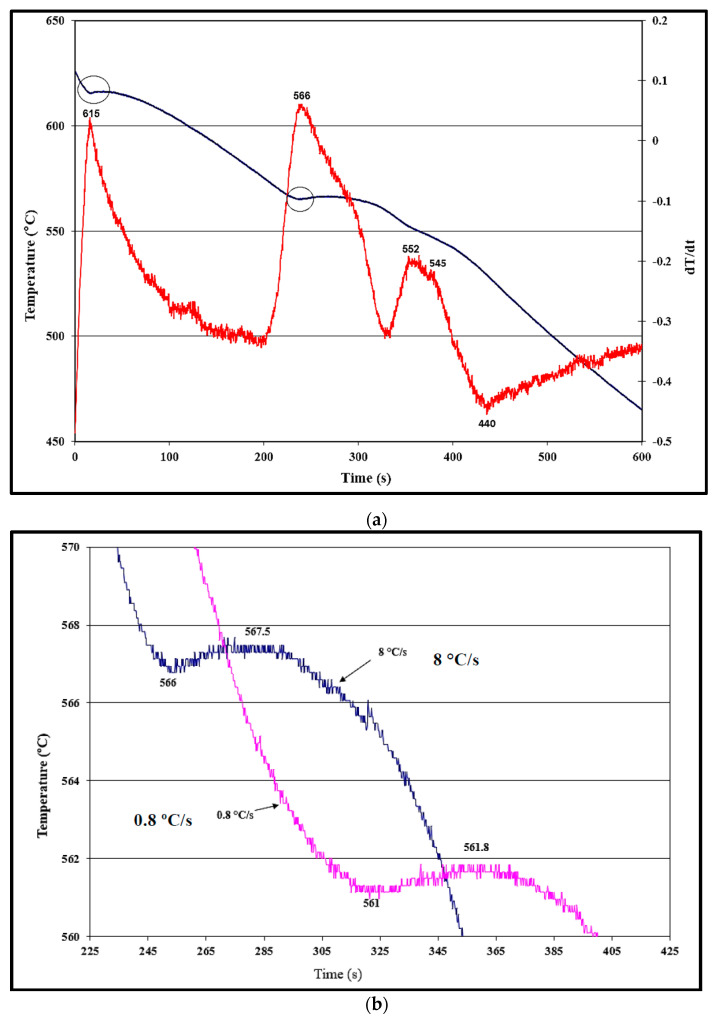

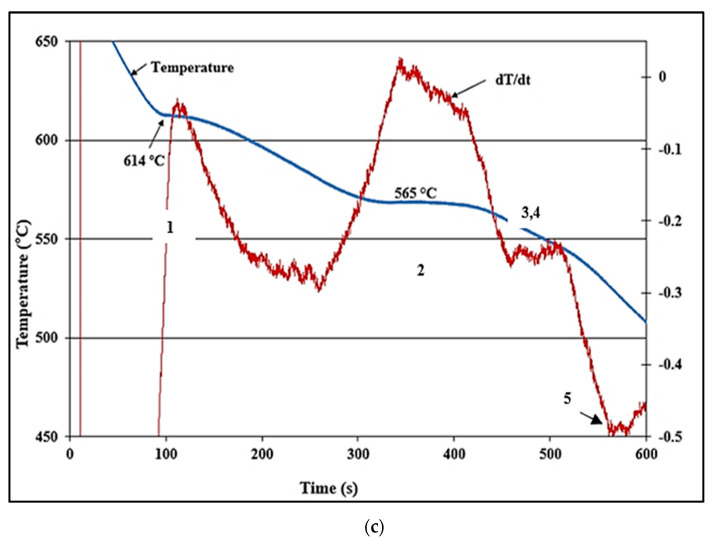
Solidification curves of the A356.2 alloy: (**a**) without a grain refiner, (**b**) effect of solidification rate, and (**c**) fully grain refined alloy.

Figure 6a depicts the solidification curve (in blue) and its first derivative (red curve) of the A356.2 alloy. The solidification rate was estimated at 0.8 °C/s. It is evident that the presence of 0.05%Ti as a grain refiner has a marginal effect of the alloy solidification characteristics in terms of the presence of clear undercooling for both α-Al and the (Al+Si) reactions. The importance of the first derivative curve is to indicate the reaction temperature [21]. Rakhmonov et al. [22] and Anjosa et al. [23] studied the effect of the solidification rate in the range of 0.3–20 K/s on the eutectic undercooling in the 356 alloy. The results showed that the characteristic parameters are influenced by the solidification rate. This statement is evidenced clearly in Figure 6b, where the undercooling of the eutectic structure remained more-or-less the same, regardless of the marked increase in the solidification rate by about an order of magnitude, 1.5 °C and 0.8 °C, respectively. Increasing the added amount of Ti to 0.18% resulted in the complete disappearance of both α-Al and eutectic undercooling, as displayed in Figure 6c. It is inferred from the first derivative that solidification started with precipitation of the α-Al network at about 616 °C (marked #1), followed by the eutectic reaction at 566 °C (marked #2), partial transformation of β-Al_5_FeSi to π-Al_8_Si_6_Mg_3_Fe [24] at 555 °C (marked #3), precipitation of Mg_2_Si at 550 °C (#4), followed by end of the solidification process at about 440 °C (#5). The total solidification time was about 450 s.

**Figure 7:**
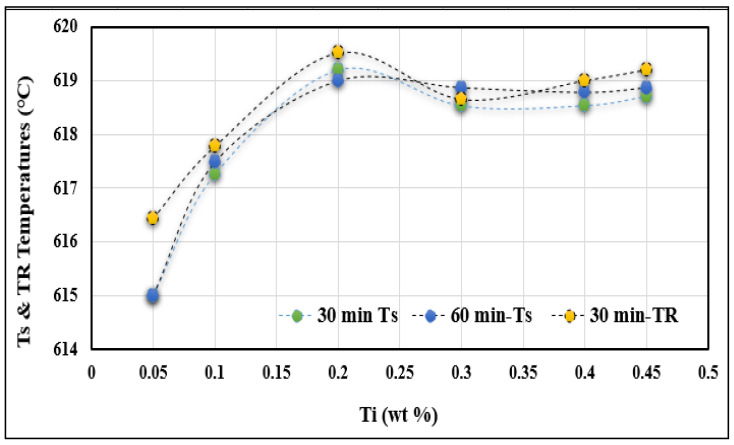
Variation in T_S_ and T_R_ as a function of added Ti; samples were poured from 750 °C after 30- and 60-min holding times.

**Figure 8:**
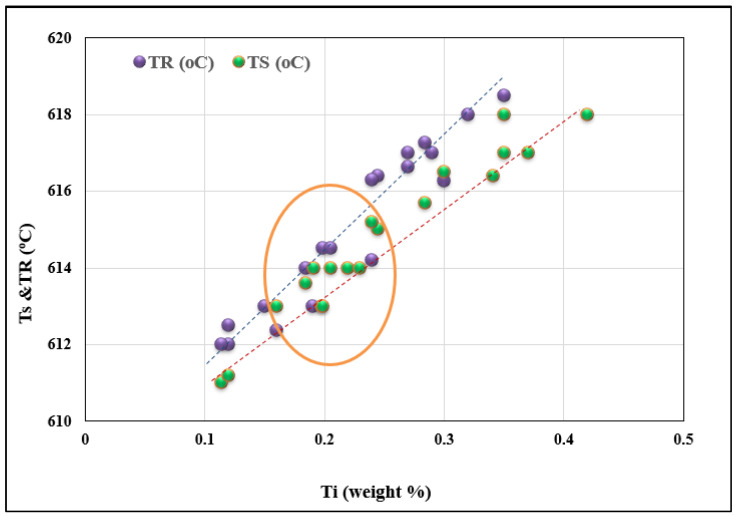
Effect of superheating at 950 °C on the T_S_ and T_R_ temperatures. The pouring temperature was 750 °C.

During casting from 750 °C without superheating, the initial increase in Ti concentration leads to a rise in undercooling and recalescence temperatures (T_S_ and T_R_). In addition, from a concentration of approximately 0.15%, a significant fluctuation in these temperatures is observed (see Figure 7). With a concentration of about 0.25%, the temperatures can oscillate between two limits (minimum and maximum). Eventually, the holding time has no significant influence on the behavior of the Ts temperatures. Superheating at 950 °C for 30 min prior to pouring from 750 °C results in a linear behavior of both Ts and T_R_ temperatures, as seen from Figure 8. However, the slope of the T_R_ temperature is slightly higher than that of the Ts temperature. In other words, Ts would reach 618 °C at 0.45% Ti, whereas it is about 0.32% Ti for the T_R_ temperature. It should be borne in mind that there is a marked clustering of Ts and T_R_ around 0.2% Ti (see circled area), which represents the optimum grain refining concentration.

The silicon dissolved in the liquid metal reacts with the Al_3_Ti particles and, similarly, some of the silicon particles react with Ti taken up by the liquid metal, due to the dissolution of the Al_3_Ti particles. The (Al,Si)_3_Ti phase existing in this system negatively affects the performance of the grain refiner by changing the parameters of the nucleation (T_S_) and recalescence (T_R_) temperatures, which are strongly influenced by the addition of Ti to the A356 alloy, in the form of a binary alloy of Al-10%Ti, as shown in Figure 7 and Figure 8. Obviously, the rates of these reactions at 950 °C are much faster at 750 °C due to the large difference in the amount of activation energies. Thus, in general, T_S_ and T_R_ temperatures increase when increasing the Ti level, independent of the superheat temperature of the liquid metal. Increasing the level of Ti to 0.25% by weight in alloys cast from 750 °C causes a dramatic drop in both the nucleation and growth temperatures (T_S_ and T_R_). As for alloys superheated at 950 °C, increasing the Ti content beyond 0.1% has the same effect on these temperatures. 

**Figure 9:**
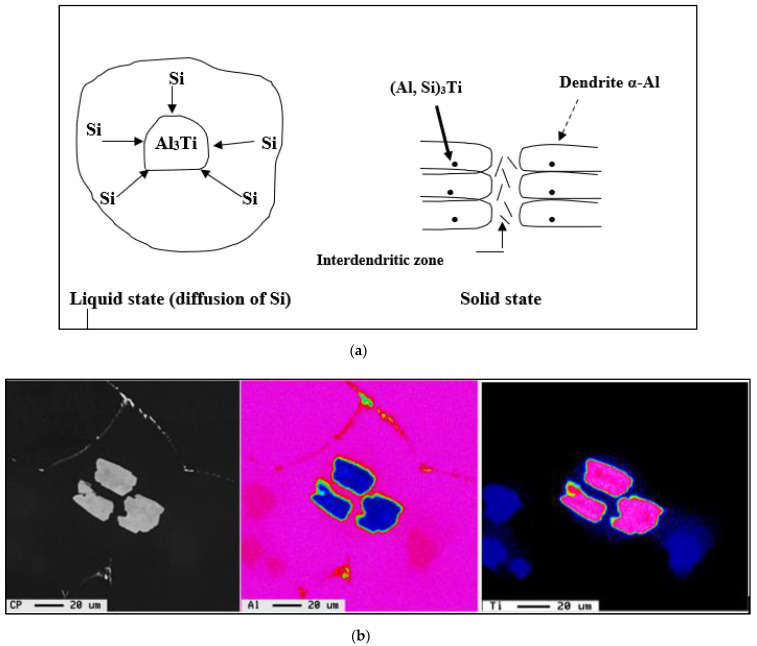
(**a**) Conversion of Al_3_Ti phase to (Al,Si)_3_Ti phase. This process is termed poisoning. The thick arrow points to precipitation of (Al,Si)_3_Ti particles within the α-Al dendritic network indicated by the broken arrow. (**b**) Left to right: Backscattered image showing precipitation of Al_3_Ti phase particles within the α-Al dendritic network, and corresponding X-ray images of Al and Ti.

As for undercooling, it also decreases with increasing Ti content up to ~0.2 wt%, and from this concentration onwards, no undercooling is observed. Similarly, the grain size also decreases from about 1854 µm (in the base alloy) to 750 µm at Ti levels of 0.2–0.25 wt% (corresponding to zero supercooling, i.e., ∆T = 0). Figure 9a summarizes the evolution of the grain size of the samples cast following two different paths. By adding the binary alloy of Al-10%Ti and in accordance with the results obtained by Youdelis and Yang [25] and those obtained by Tahiri et al. [26,27], Al_3_Ti intermetallics are transformed into ternary compounds of the type (Al,Si)_3_Ti.

According to the law of conservation of matter, the percentage by weight of total Si in the alloy (=7% by weight) is the sum of the percentage of Si present in the intermetallics (Al,Si)_3_Ti and the percentage of Si existing in the matrix. The schematic diagram in Figure 9a shows how Si is inserted into Al_3_Ti particles. During the liquid state, these last particles are in contact with Si. Because of the diffusion of this element, Si joins the Al_3_Ti particles to form compounds of the (Al,Si)_3_Ti type. These compounds constitute nucleation sites for the pre-eutectic α-Al phase. Obviously, these sites are located in the dendritic cells and not in the inter-dendritic zones, as shown in Figure 9a. The Si gradient at the walls of the (Al,Si)_3_Ti particles is strong, as the diffusion of Si is important. The further one moves away from the interface between the particle and the Al matrix, the percentage of Si decreases before it stabilizes. Figure 9b depicts the precipitation of Al_3_Ti phase particles within the α-Al network.

**Figure 10:**
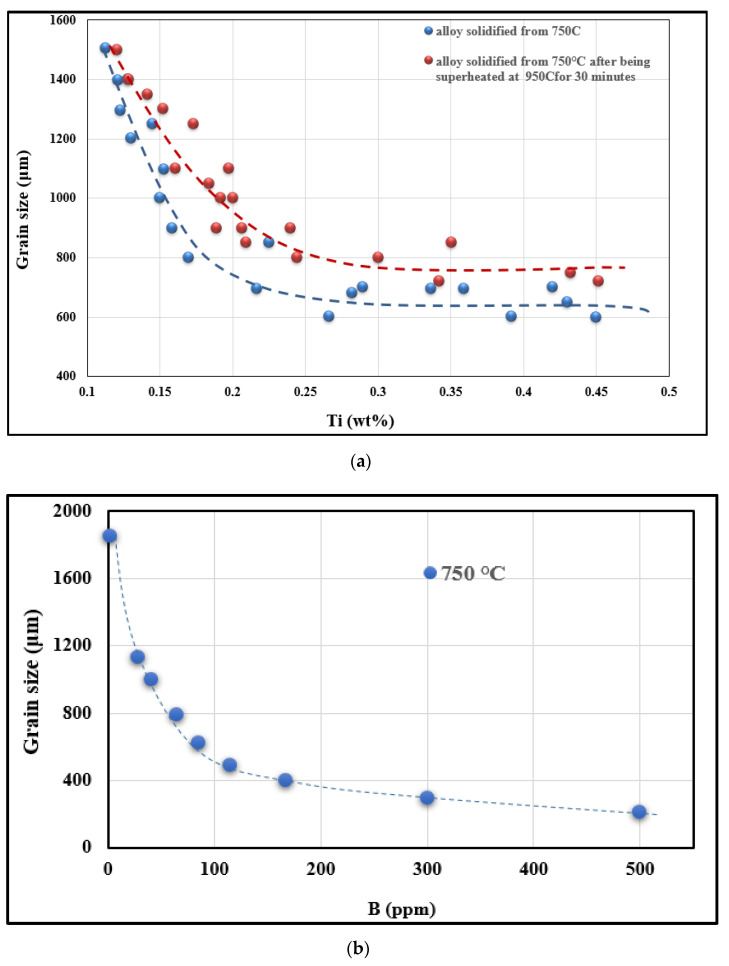
(**a**) Effect of the added Ti content and casting procedure on the alloy grain size. (**b**) Effect of the added B content on the alloy grain size.

When the Al-4%B master alloy is added to the liquid Al alloy with a low Ti concentration, i.e. 0.5%Ti, the B atoms that come from the melting of the master alloy will react with the Ti atoms in the liquid bath to form TiB_2_ type particles. The reaction can be expressed as: Ti + 2B → TiB_2_. According to thermodynamic calculations, the change in Gibbs free energy of this reaction is ΔG = −73,381 + 38.996T. ΔG is negative at 720 °C (it is equal to −34,657.97 kJ/mole). Thus, the reaction is a spontaneous process around 720 °C and the TiB_2_ particles formed in the reaction should be very fine. TiB_2_ particles have a high enthalpy of formation, which is around 326.41 kJ/mole [28]; hence, they are stable in the molten metal. Figure 10a shows the variation in grain size with Ti content in the A356 alloy. Figure 10b shows the variation in grain size as a function of B content. It is obvious that at a lower level of B, the grain size of alloy A356 decreases rapidly with increasing B content.

At a low Ti content, the Al_3_Ti particles dissolve rapidly and present Ti as a dissolved body in liquid aluminum. TiB_2_ particles, on the other hand, are quite stable in the liquid aluminum alloy, and it is these particles that act as nucleation substrates for the primary α-Al phase. However, if the liquid metal remains steady after the addition of the master alloy, the effect of grain refining weakens over time [15]. As defined by Stokes’ law [23], the size and density of the particle have a significant impact on the time required for the particle to descend to a distance S. According to Stokes’ law [14,29], a low density and small size of the grain refining particles should result in a longer settling time, and thus the grain refining effectiveness weakens as follows:

(3) $$t=\frac{18\mu }{gd^{2}(\rho_{P}-\rho_{L})}S$$

where *t* is the settling time; *µ* is the viscosity of the liquid; *g* is the gravitational acceleration (*g* = 9.80 m/s^2^); *d* is the diameter of the particle; *ρ_P_* and *ρ_L_* are the densities of the particle and of the liquid, respectively; and *S* is the displacement of the particle.

The role of titanium diboride, TiB_2_, in the grain refining process is a matter of different interpretations. Valid information on this role was obtained from the inoculation experiments performed in the work of Khalifa et al. [18]. The introduction of TiB_2_ particles into molten Al alloys was considerably facilitated due to their good wettability with aluminum. The uniform distribution of these particles in the alloy is the key to a successful evaluation of their role in the development of the solidification microstructure. To achieve or maximize this kind of distribution, pouring should be done as soon as the mechanical stirring is completed. Furthermore, the stirring should be maintained at maximum until the pouring time. It has also been proven that TiB_2_ particles have a high stickiness or adhesion coefficient. The TiB_2_ particles tend to form clusters in the liquid metal during the liquid stage. The decay phenomenon related to grain refiners in molten alloys can thus be attributed to the agglomeration of TiB_2_ particles. 

### 3.2. Macro and Microstructure Characterization

**Figure 11:**
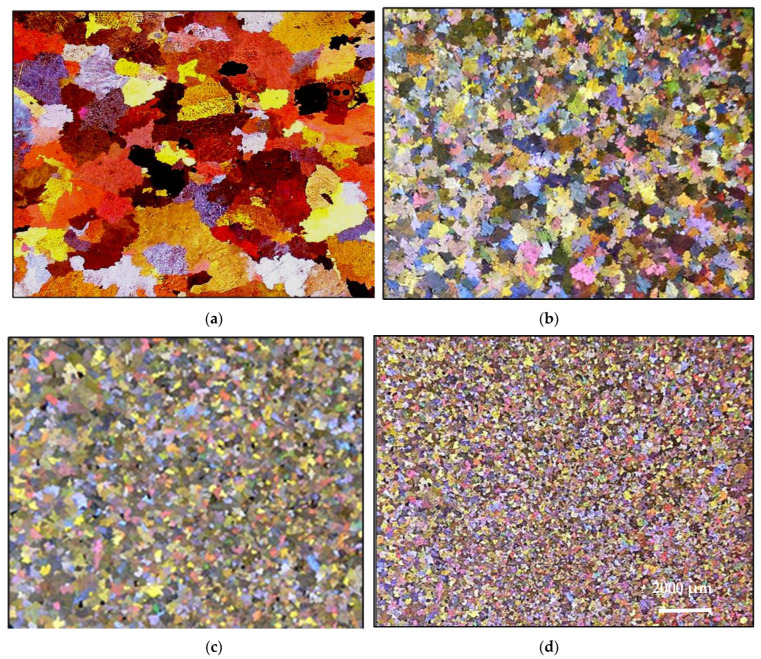
Effect of Ti and B addition of the A356 alloy with grain size of: (**a**) 1800 µm (0.05%Ti), (**b**) 850 µm (0.12%Ti), (**c**) 450 µm (0.22%Ti), and (**d**) 200 µm (500 ppm B).

**Figure 12:**
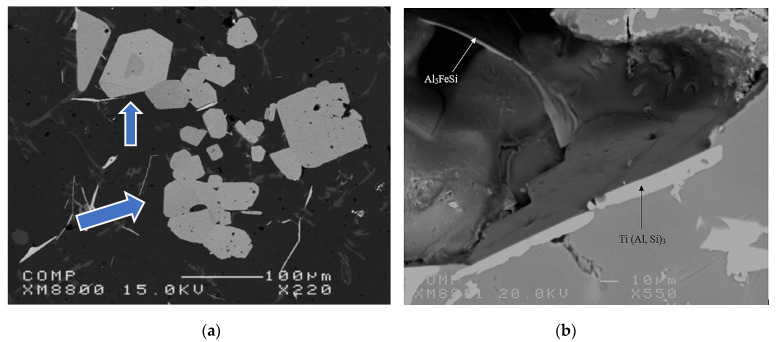
(**a**) Backscattered electron image of (Al,Si)_3_Ti platelets (arrowed blue) in the 0.35%Ti-containing A356 alloy and (**b**) a high magnification micrograph of the (Al,Si)_3_Ti platelets.

**Figure 13:**
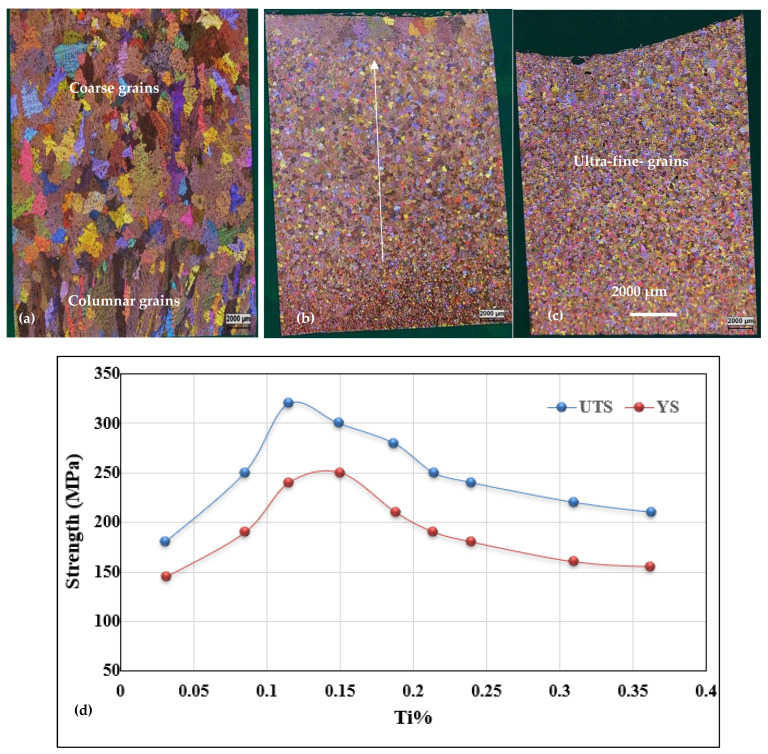
Macrostructure of the A356 alloy with (**a**) no grain refining; (**b**) addition of 0.15%Ti- note gradual decease in grain size as indicated by the white arrow; (**c**) 500 ppm B, casting was made in a permanant metallic mold (dimensions: 0.5 cm × 8 cm × 10 cm); (**d**) strength−Ti% relationship.

**Figure 14:**
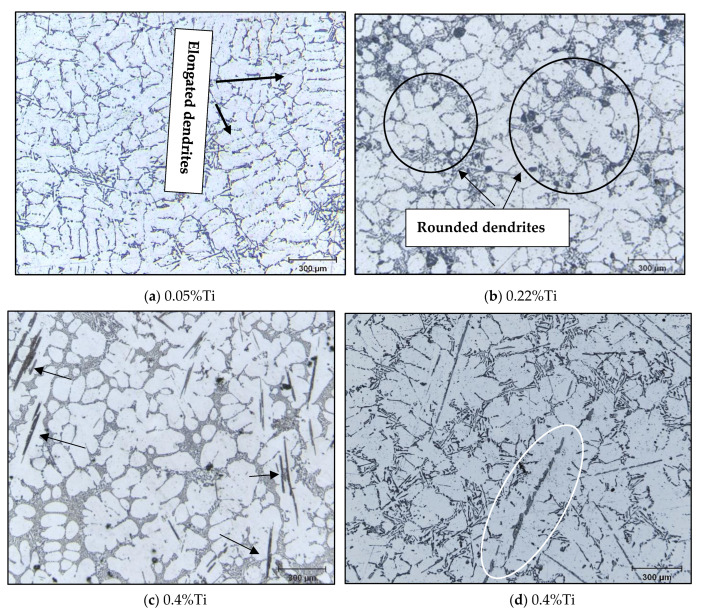

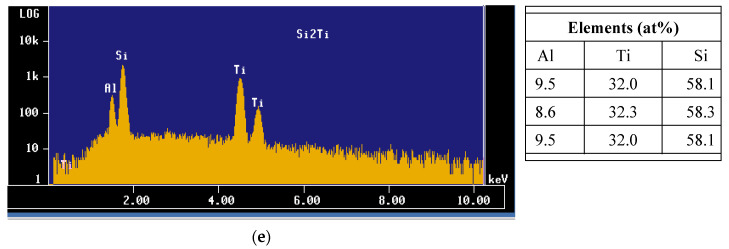
Optical microstructures of A356.2 alloy at different addition of Ti: (**a**) as received alloy (0.05%Ti); (**b**) 0.22%Ti cast from 750 °C (30 min stirring); (**c**) 0.4%Ti with no superheating; (**d**) 0.4%Ti superheating at 950 °C prior to casting from 750 °C; (**e**) EDS corresponding to (**e**) revealing strong peaks of Al, Si, and Ti-inserted table, indicating the actual composition measured at three spots. Black arrows in (**c**) point to (Al,Si)_3_Ti platelets within the α-Al network.

Figure 11 gives an overview of the grain size when the A356 alloy is treated with two grain refiners. In the absence of any addition, the grain size is about 1500 µm in the base alloy (Figure 11a). Figure 11b clearly reveals the poisoning effect caused by Ti−Si interaction, leading to the formation of (Al,Si)_3_Ti. As a result, the average grain size is about 850 µm. Thus, in order to overcome this situation, the Ti content was increased to 0.22%, resulting in a grain size of around ~450 µm (Figure 11c). A minimum value was obtained when the boron-based refiner (Al-4%B) was used, reducing the grain size to 200 µm (Figure 11d), corresponding to 500 ppm of B. Excess addition of Ti or B has no effect in reducing the aluminum grain size. On the contrary, access to Ti or B can lead to deleterious effects on the microstructure of the alloy, and consequently have an impact on its mechanical properties. Figure 12 exhibits the effect of superheating at 950 °C on the size of the (Al,Si)_3_Ti platelets. From an industrial point of view, grain refiners have a significant effect on the uniform distribution of grain size and shape within the casting, as illustrated in Figure 13, leading to superior mechanical properties [30,31]. Figure 13d depicts the relationship between the added Ti and the alloy strength obtained. As can be observed, the maximum strength was achieved when Ti was added in amounts of 0.15%, followed by a gradual decease with increasing the amount of Ti added due to the simultaneous increase in the volume fraction of the precipitated Al_3_Ti in the form of platelets, as shown in Figure 12 and Figure 14.

Depending on the type of the added master alloy, the α-Al dendritic phase is characterized by a particular morphology. The A356 base alloy shows dendrites with an elongated shape with a non-uniform distribution, as shown in Figure 14a. In the case of the addition of Al-10%Ti, the dendrites exhibit a tendency to form rosettes (as shown in Figure 14b, see areas circled in black). The morphology of the (Al,Si)_3_Ti intermetallics can have a plate shape when the alloy is heated to 750 °C or a dendritic shape when the superheating temperature is 950 °C (see Figure 14b,d,e). As may be seen in Figure 14c, the (Al,Si)_3_Ti intermetallics act as sites for the nucleation of the α-Al network phase, as denoted by the black arrows. In addition, increasing the amount of added Ti increases the volume fraction of the dendrites that transformed into a rosette shape. The volume fraction of these intermetallics increases with the amount of the added Al-Ti alloy to the liquid metal. However, their size does not show a similar trend, which is mainly related to their original size in the master alloy used (i.e., rods or waffles).

**Figure 15:**
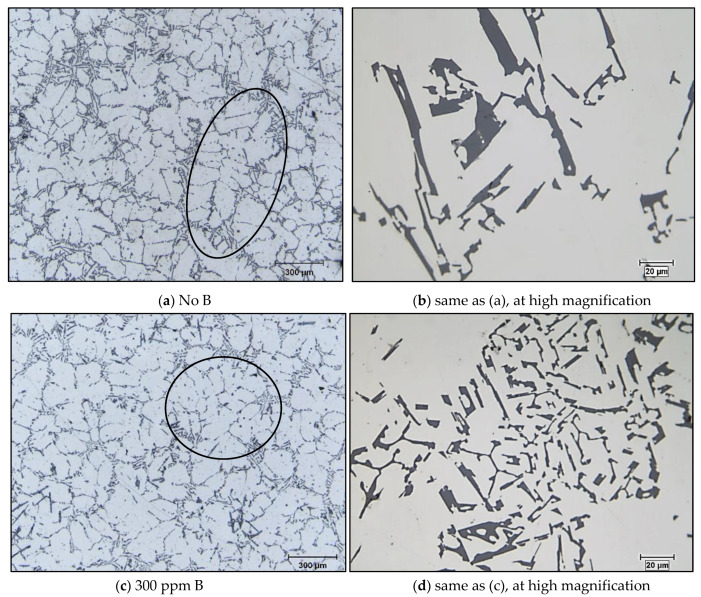
Optical microstructure of the A356 base alloy following treatment with B: (**a**) as received alloy showing elongated dendrites (see black circle); (**b**) acicular eutectic Si particles in (**a**); (**c**) 300 ppm B showing rounded dendrites (see black circle); (**d**) partially modified Si particles in (**c**).

As the net interfacial energy between the TiB_2_ particle and the α-Al phase is larger than that between the TiB_2_ particle and liquid aluminum, and the lattice difference between the TiB_2_ particle and the α-Al phase is also very large [14,29], during the solidification of liquid aluminum, the TiB_2_ particles tend to be pushed to the grain boundaries, and are not present in the center of the α-Al phase. Thus, TiB_2_ particles alone cannot usually act as active nuclei for aluminum according to several references [15,32]. When refiner with B is added to the liquid metal, the α-Al phase takes a rounded structure (Figure 15c). The change in dendrite morphology leads to a decrease in the apparent viscosity of the alloy. The circled areas in the optical micrographs shown in Figure 15 clearly illustrate that the primary α-Al phase exhibiting an elongated dendritic structure in an untreated specimen (Figure 15a) changes to an equiaxed morphology with the addition of boron (Figure 15c). The eutectic is also affected by this treatment, as the eutectic Si particles partially transformed into a fibrous form (see Figure 15d). 

### 3.3. A390.1 Alloy

**Figure 16:**
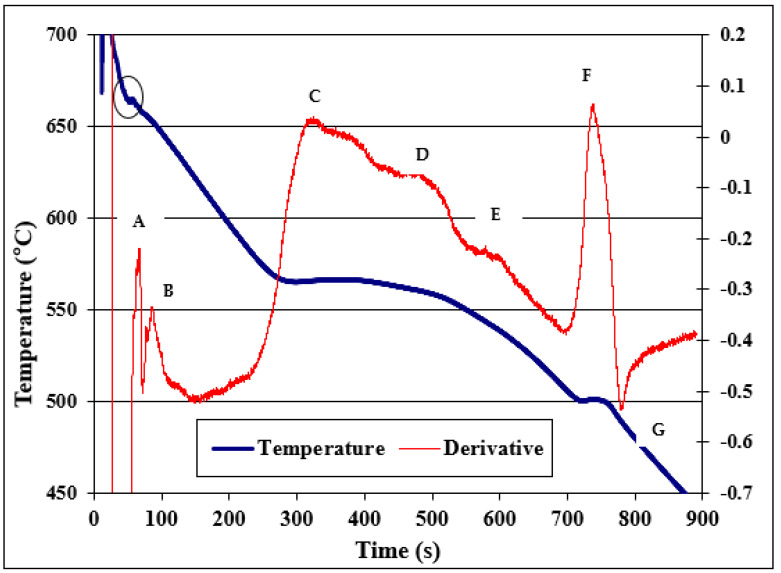
Thermal analysis curve and first derivative of an A390 alloy (17%Si) as cast at a solidification rate of ~0.8 °C/s in a preheated graphite mold. Note undercooling circled at the top left on the blue solidification curve. The letters A to G correspond to the reactions listed in Table 2.

**Figure 17:**
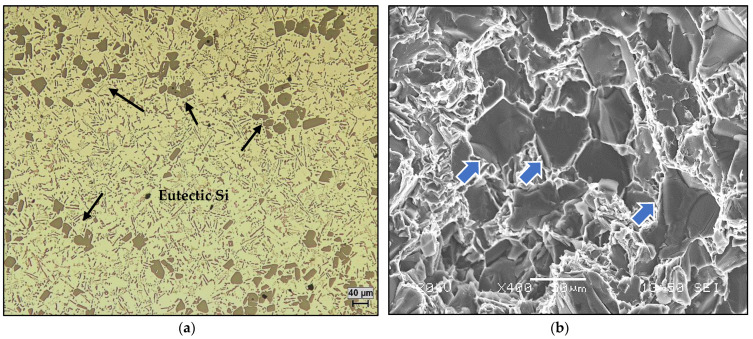
(**a**) Optical microstructure of the A390.1 alloy solidified at 0.8 °C/s, (**b**) fracture surface of same alloy solidified at 8 °C/s. Black and blue arrows in (**a**) and (**b**), respectively, point to the primary Si particles.

This alloy is used either for pressure casting or for permanent mold casting. The high silicon content (17% Si) gives the liquid metal good fluidity and the many Si particles also result in a high wear resistance and low thermal expansion. The high concentration of copper (Cu, 4 to 5%) and magnesium (Mg, 0.50 to 0.60%) makes this alloy heat treatable, thus providing excellent properties at elevated temperatures. Typical uses for this alloy are found in engine blocks, cylinder heads, and compressors. Thermal analysis was applied to determine the thermal characteristics, the fraction of the solid, and the amount of heat released during the solidification process. The thermal analysis curve relating to the hypereutectic alloy A390 is given in Figure 16, and Table 2 lists the corresponding reactions that take place during solidification. Based on the phase diagram of the Al-Si binary system, during cooling, the liquid metal gives the first precipitation relating to the deposition of primary Si particles. The latter take the form of very coarse blocks with a size varying between 100 and 150 µm, as shown in Figure 17a. Figure 17b reveals the fracture surface of a test bar pulled to fracture, showing the adherence of primary Si with the surrounding aluminum matrix.

**Figure 18:**
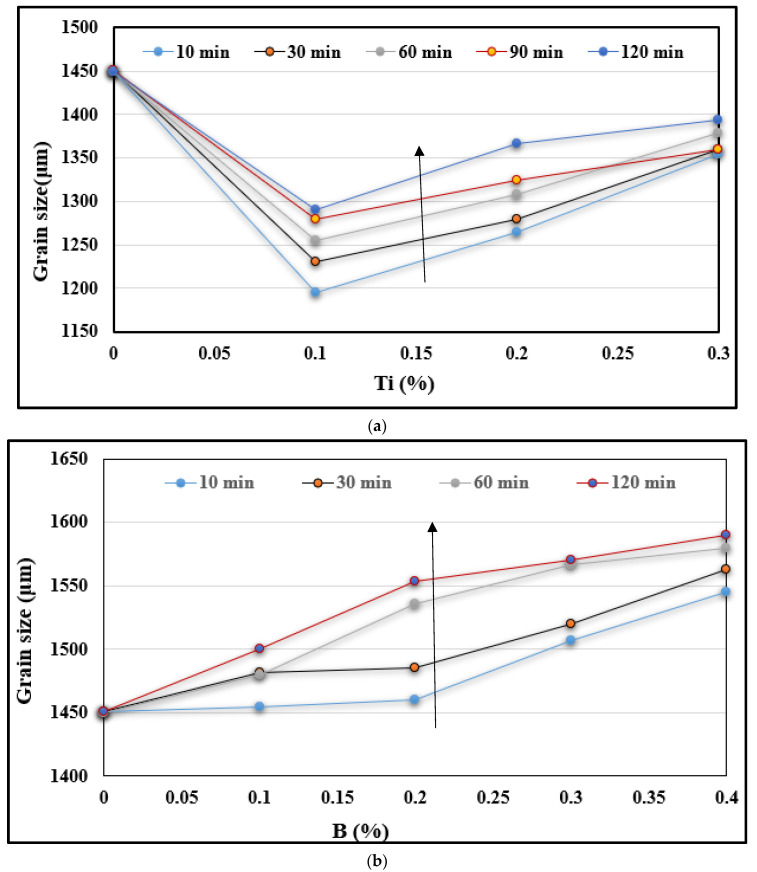
Effect of (**a**) Ti and (**b**) B addition on the grain size of the A390 alloy. Note the increase in the grain size with the increase in holding time, as noted by the vertical black arrows in each case.

The evolution of the grain size in hypereutectic alloy 390 presented in Figure 18a,b depicts the respective effects of Ti and B additions on the alloy grain size. As may be observed from Figure 18a, in the base alloy (alloy that has not undergone any treatment), the grains are coarse and their size reaches a value ranging from 1450 to 1600 µm as the maximum value. When 0.1% Ti in the form of Al-10%Ti is added to this base alloy, the grain size is only reduced to about 1200 µm, especially when the liquid bath is held just for 10 min before being cast. This reduction in size is equivalent to 25% compared with 67% in the case of the addition of 0.1% Ti to the hypoeutectic alloy A356. We also note a loss of reduction in the size of the grains with the progressive increase in Ti content and the extension of the holding time of the liquid metal. The longer the latter is maintained, the more particles of (Al,Si)_2_Ti type tend to form, which results in a more acute weakening of grain refining. 

The maintenance of the liquid metal has a negative impact on the reduction in this size, as the latter increases according to the increase in the maintenance time of the liquid bath. Thus, even if the Al-4%B master alloy content increases, the grain size does not undergo a large change and it is almost constant for a given holding time, as seen from Figure 18b. As the Al-10%Ti grain refiner generates two nucleation sites (Al_3_Ti and TiB_2_) for the pre-eutectic α-Al phase, these locations are strongly influenced by the high affinity between Ti and Si, leading to the formation of TiSi_2_. It is evident from Figure 18 that increasing the holding time increases the Ti-Si, Si-B and Ti-B interactions (poisoning), resulting in an increase in the alloy grain size.

**Figure 19:**
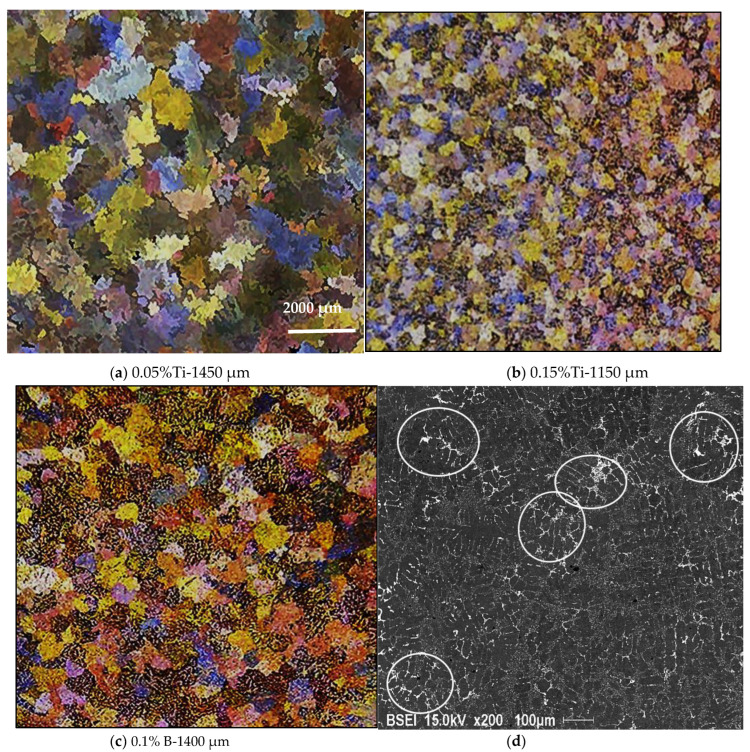
(**a**,**b**) Variation in the A390.1 alloy grain size as a function of the added (**a**,**b**) Ti, and (**c**) B content; (**d**) distribution of Al_2_Cu phase particles in the as-cast base alloy, see white circled areas.

Figure 19 reveals the shape of the grains in the hypereutectic alloy A390 with different refiner contents. The grain boundaries cannot be distinguished clearly from each other. This difficulty is mainly due to the high Si content (~17% Si) as well as the presence of Cu in a large percentage (~4.5%). The latter reacts very quickly with the acids used for chemically attacking the sample surface in order to reveal the macrostructure of the alloy studied.

## 4. Conclusions

Based on the results of the thermal analysis and a study of the macrostructure and the microstructure of the A356.2 and A390.1 alloys investigated, the following conclusions may be drawn.

1. Undercooling (T_S_) and recalescence (T_R_) temperatures increase with the initial increase in Ti concentration. At approximately 0.25%, a rapid decrease in these temperatures is observed.
2. The minimum grain size is achieved when the Ti concentration exceeds about 0.20%.
3. Superheating has a negative effect on the structure of the alloy. In general, high temperature casting leads to a coarse grain size.
4. The Al-4%B master alloy shows remarkable grain refining effectiveness in comparison with Al-10%Ti. The residual Ti in the A356 alloy reacts with boron B to form TiB_2_, which subsequently acts as an active seed for the α-Al phase. Grain refining is achieved primarily with TiB_2_ rather than AlB_2_, or both, depending on the Ti content in the alloy.
5. The Al-10%Ti master alloy is rich in Al_3_Ti particles. Once added to the molten alloy, these particles convert into other intermetallics (Al,Si)_3_Ti.
6. The term “poisoning”, reported frequently in various studies on grain refining to explain the loss or weakening of the effectiveness of Al_3_Ti nucleation sites, is misused, as these nucleation sites undergo a phase transformation of the type Al_3_Ti → (Al,Si)_3_Ti → (Al,Si)_2_Ti, which reduces their efficiency to nucleate the pre-eutectic α-Al phase. Hence the removal of the term “poisoning” is sought when describing weakening of the grain refiner in such cases.
7. In the A390.1 alloy with a high Si content (~17%), the grain size varies between 1450 µm and 1600 µm maximum vs. 1850 µm for A356.2 alloy for the base alloys. This suggests that Si plays the role of a grain refiner in the absence of the usual refiners.

## Figures and Tables

**Table 1:** Composition of the A356.2 and A390.1 alloys used.

Alloy	Al	Si	Cu	Mg	Fe	Mn	Zn	Ti
A356.2	bal.	6.78	0.02	0.33	0.11	0.04	0.04	0.07
A390.1	bal.	17.30	4.33	0.54	0.32	0.06	0.06	0.07

**Table 2:** Expected reactions in the A390.1 alloy.

	Reactions	Temperature (°C)
A	Primary Si	670
B	Developpement of α-Al network	561
C	Liq. → Al + Si + Al_5_FeSi	575
D	Liq. → Al + Si + Mg_2_Si	555
E	Liq. + Mg_2_Si → Al + Si + Al_2_Cu + Al_5_Mg_8_Cu_2_Si_6_	512
F	Liq. → Al + Al_2_Cu + Al_5_Mg_8_Cu_2_Si_6_	502
G	End of splidification	490
Total solidification time: approximately 750 s		

## Data Availability

Data will be made available upon request.
